# A case of benign periosteal chondroma seeding into humeral medullary bone via percutaneous needle biopsy tract

**DOI:** 10.1259/bjrcr.20150104

**Published:** 2015-05-07

**Authors:** J P Learmont, G Powell, J Slavin, M Facey, M Pianta

**Affiliations:** ^1^Peninsula Health, Frankston, VIC, Australia; ^2^St Vincent’s Hospital Melbourne, Fitzroy, VIC, Australia

## Abstract

We report an occurrence of periosteal chondroma seeding into the medulla of humerus via percutaneous needle biopsy tract. To our knowledge, this is the first described case of benign cartilage tumour biopsy tract seeding in the literature. We discuss the clinical, radiological and histological features of periosteal chondroma, as well as the diagnostic challenges associated with distinguishing this entity from periosteal chondrosarcoma. Finally, we briefly discuss the safety of imaging-guided percutaneous needle biopsy and methods to minimize the risk of iatrogenic tumour seeding.

## Clinical presentation and investigation findings

**Figure 1.**
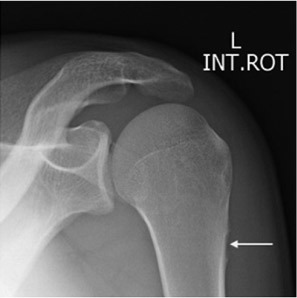
An 18-year-old male presents with proximal arm pain. Anteroposterior radiograph of the proximal left humerus at initial presentation demonstrating cortical saucerization of the proximal metaphysis (arrow).

**Figure 2.**
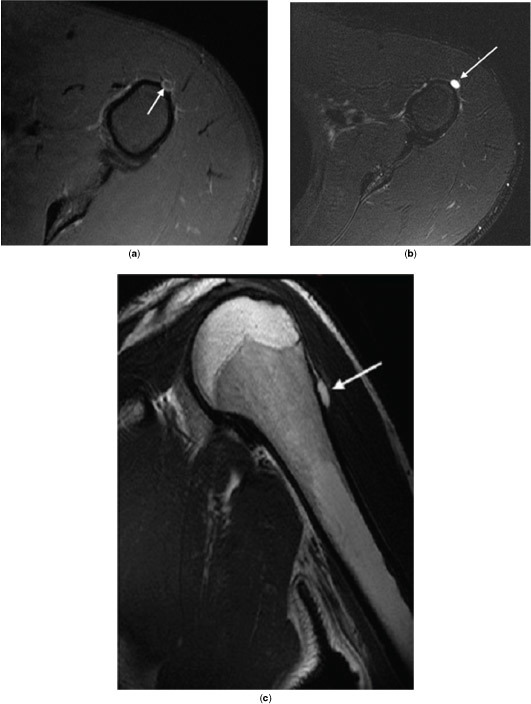
MR scans performed at initial presentation. (a) Axial *T*_1_ fat-saturated post-contrast image demonstrating periosteal lesion rim with low-grade enhancement (arrow). (b) Axial *T*_2_ fat-saturated image demonstrating a hyperintense periosteal lesion (arrow) of the left humerus cortex with saucerization. (c) Coronal *T*_2_ fast spin echo image demonstrating hyperintense periosteal lesion of the left humerus (arrow). Absence of bone marrow invasion is observed.

An 18-year-old previously well male presented to his local practitioner with a several-month history of mild left proximal arm pain. There was no preceding trauma. Shoulder ultrasound was normal, including the rotator cuff. Plain radiograph demonstrated saucerization of the humerus cortex at the proximal metaphysis (Figure 1). CT scan demonstrated an intracortical lesion with saucerization but no associated soft tissue mass or cortical breach. MR imaging showed a well-circumscribed, homogeneous juxtacortical lesion measuring 6.5 × 4.5 × 13 mm (anteroposterior × mediolateral × craniocaudal) with scalloping of the adjacent cortex. *T*_1_ weighted sequences showed the lesion to be isointense relative to adjacent muscle. There was low-grade peripheral contrast enhancement (Figure 2a). *T*_2_ weighted sequences showed central hyperintensity, compatible with cartilage (Figure 2b,c). All sequences demonstrated a focus of signal hypointensity within the lesion, likely representing calcification. There was no bone marrow or adjacent soft tissue oedema. Thallium scan was negative for metabolic activity. Periosteal chondroma was considered the most likely diagnosis given the absence of medullary invasion and small lesion size; however, periosteal chondrosarcoma could not be excluded owing to the presence of pain, necessitating biopsy.

**Figure 3.**
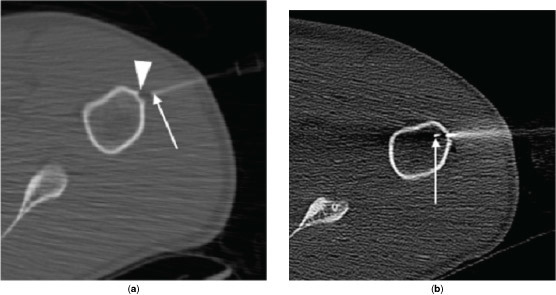
CT-guided core needle biopsy at initial presentation. (a) Axial CT scan, bone algorithm, of the left humerus during first pass biopsy showing location of Bard Truguide 17 G × 7 cm coaxial needle (Bard Biopsy Systems, Tempe, AZ) (arrow). Absence of cortical breech is observed (arrowhead). (b) Axial CT scan, bone algorithm, of the left humerus during second pass biopsy showing AprioMed Bonopty 15 G × 16 cm biopsy needle (Apriomed AB, Uppsala, Sweden) breaching the cortex with needle tip (arrow) located just within the medulla.

CT-guided core needle biopsy of the superficial cortical portion of the lesion was performed using a coaxial technique with Bard TruGuide 17 G × 7 cm and Bard Magnum 18 G × 10 cm biopsy needle (Bard Biopsy Systems, Tempe, AZ) (Figure 3a). The tissue sample was non-diagnostic, yielding skeletal muscle only. After discussion with the referring Sarcoma Multidisciplinary team, a second pass biopsy was performed using AprioMed Bonopty 14  G × 9.5 cm penetration set and AprioMed Bonopty 15 G × 16 cm biopsy set (Apriomed AB, Uppsala, Sweden). The biopsy needle was bored through the humeral cortex to obtain an adequate tissue sample and ensure there was no subcortical involvement of the lesion (Figure 3b). Histological analysis with haematoxylin and eosin stain revealed chondroid tissue with loose myxoid stroma and evenly scattered chondrocytes with no atypia. Immunohistochemistry was not necessary for diagnosis in this case. Consensus agreement for periosteal chondroma was made at a soft tissue tumour multidisciplinary meeting. Conservative management was advised.

## Follow-up

**Figure 4.**
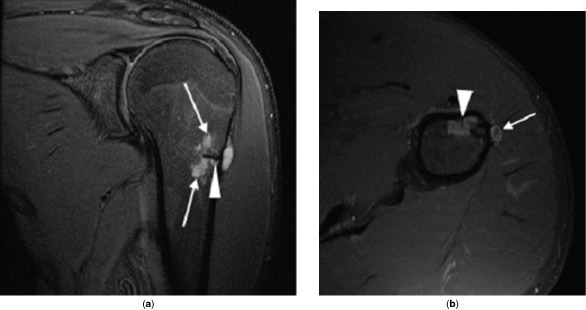
MR images obtained 10 months after those seen in Figure 2, to investigate the complaint of worsened pain. (a) Coronal *T*_2_ fat-saturated image demonstrating the presence of hyperintense intramedullary lobulated mass (arrows) deep to the low signal, linear needle biopsy tract (arrowhead). (b) Axial *T*_1_ fat-saturated post-contrast image demonstrating the original juxtacortical lesion (arrow) as well as cartilage enhancement in the medullary cavity (arrowhead).

The patient re-presented 10 months later owing to worsening pain. MRI showed the original lesion had developed a cartilaginous, multilobulated component, invading through the cortical biopsy tract into the medulla (Figure 4a,b). Repeat biopsy was performed via the original tract, taking samples of surrounding abnormal bone. Histology demonstrated bone that was partially replaced by low grade, bland chondroid material, with a hyaline matrix, sparse chondrocytes and no atypia.

## Treatment

**Figure 5.**
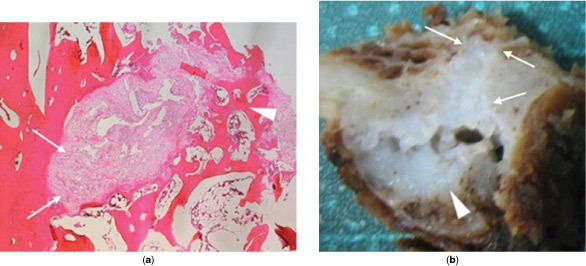
Postoperative tissue specimens. (a) Microscopic view with haematoxylin and eosin stain demonstrating benign chondroid material (arrows) extending into medullary bone (arrowhead). (b) Macroscopic view demonstrating chondroid lesion (arrowhead) with needle-like extension into adjacent bone cortex and medulla (arrows).

Wide-margin surgical resection was performed, confirming the presence of periosteal chondroma with extension through a cortical deficit into the underlying medulla (Figure 5a,b). The medullary component was treated with phenol and filled with bone cement. This approach was favoured over en block resection with auto- or allograft reconstruction, as the patient had a history of heavy smoking, which could potentially complicate wound healing.

## Outcome

Postoperative recovery was uncomplicated. The patient was reviewed 3 months postoperatively and had no further left proximal arm pain.

## Discussion

We present a unique case of intramedullary seeding of periosteal chondroma following iatrogenic trauma from percutaneous core needle biopsy. Invasion of a cartilaginous mass, such as periosteal chondrosarcoma, into an extra-anatomical compartment is a characteristic previously believed to be exclusive to malignant lesions.^1,2^ Differentiation of periosteal chondroma from low-grade periosteal chondrosarcoma is challenging, as there is considerable overlap of patient age, symptomatology, radiological and histological features between these entities.^1–3^ However, the prognosis and hence management of these lesions differs. Periosteal chondromas are benign and usually curable with local excision; recurrence is rare.^1–3^ Recurrence of periosteal chondrosarcoma following local excision with complications, including pulmonary metastasis and death, has been reported by multiple authors, thus wide excision or amputation is advised.^2,4^

Periosteal chondromas are benign cartilaginous growths originating from the deep surface of long bone periosteum. Together with periosteal chondrosarcoma, these lesions represent approximately 1% of all bone neoplasms.^1,2^ Periosteal chondromas arise from the metaphyseal region of long bones, with the most common sites being humerus, femur, tibia and phalanges.^3,5^ Macroscopically, these lesions appear as a lobulated cartilaginous mass localized to the bone cortex invested by intact periosteum.^1^

Periosteal chondroma commonly presents with pain; however, it typically has a gradual onset of several years and is usually associated with a mass or previous traumatic episode.^1,2^ Pain in the absence of other symptoms accounts for only 25% of presentations.^1,2^ Onset of pain of less than 1-year duration as seen in this case is a common feature of periosteal chondrosarcoma, present in almost 50% of patients.^4^ Regarding the assessment of cartilage tumours, Bauer et al^1^ suggest that isolated pain in the absence of preceding trauma may be owing to the aggressive biological behaviour of a lesion such as periosteal chondrosarcoma.

Periosteal chondromas are usually well marginated on radiographic evaluation with sharply demarcated saucerization of adjacent bone cortex, often demonstrating adjacent cortical destruction lending a scalloped appearance; in some cases, the cortical bone may be seen to enclose the lesion with partially overhanging edges.^1,2,5^ Periosteal chondrosarcomas more often demonstrate poorly defined lytic margins without appreciable mass effect on adjacent cortex.^2^

On *T*_1_ weighted MR sequences, periosteal chondromas demonstrate hypointense to isointense signal relative to muscle.^5^*T*_2_and *T*_2_* weighted sequences characteristically demonstrate the lesion to be hyperintense relative to fat, attributable to the high water content of hyaline cartilage; a peripherally based hypointense lining correlating to intact periosteum is also usually seen.^5^ Bone marrow or surrounding soft tissue oedema are rare features.^3, 5^ Focal areas of signal hypointensity on all image acquisition sequences likely reflect internal tissue matrix calcification, notable in approximately 50% of lesions.^5^ Following the administration of gadolinium-diethylenetriamine pentaacetic acid Gd DTPA contrast, Woertler et al^5^ described enhancement of the lesion periphery in 100% of cases.

It is recognized that the histological characteristics of periosteal chondroma can vary from bland cartilage to low-grade cancer, whereas chondrosarcomas have been shown to always meet the histological criteria of a malignant diagnosis.^2^ Histological analysis of periosteal chondromas demonstrates lobules of immature hyaline cartilage with small chondrocytes present. Additionally, these lesions may feature cellular atypia, hypercellularity, multinucleation, hyperchromatic nuclei or myxoid change in the matrix, making them difficult to distinguish from low-grade chondrosarcoma.^1,2^

Discerning periosteal chondroma from periosteal chondrosarcoma can be difficult. Lesion size can be useful in differentiating these entities. The average size of periosteal chondroma has been shown to vary from 2.2 to 2.8 cm, with no lesion greater than 7 cm being reported in the literature.^1–3,5^ Multiple authors have demonstrated the size of periosteal chondrosarcoma to measure greater than 5.5 cm.2,3 To date, there has been no reported case of periosteal chondrosarcoma measuring less than 3 cm in size.^2,3^

Periosteal chondroma was previously thought to be distinguishable from periosteal chondrosarcoma by the absence of medullary invasion.^1,2^ This characteristic was recently challenged by Robinson et al^3^ in a study correlating the histological and radiological findings of cartilaginous periosteal lesions, where medullary invasion of periosteal chondroma was reported on CT and MR imaging in 4 out of 22 patients. The reporting osteopathologist was blind to the radiological findings in each case. Given the recommendation from several authors that differentiation of periosteal chondroma from low-grade periosteal chondrosarcoma be made by collaborating clinical, radiological and pathological data rather than based solely on histopathology, it is possible these four cases may have been reported as chondrosarcoma if the osteopathologist had access to the radiological studies.^1,2^

The lesion identified in our study did have well-defined margins and measured less than 3 cm in maximal dimension, supporting the diagnosis of periosteal chondroma. However, the recent onset of pain in the absence of trauma could have been owing to a low-grade periosteal chondrosarcoma. At our institution, all radiologically detected lesions presenting with characteristics that can be reasonably attributed to a malignant process such as atraumatic pain are further investigated with biopsy. Had the biopsy result demonstrated a malignancy, neoadjuvant chemo- and radiation therapy would have been considered with en block resection of the lesion.

To our knowledge, intramedullary seeding of a benign cartilaginous tumour, as seen in this case, has not been reported in the literature, although seeding of sarcoma following core needle biopsy is a rare but recognized complication.^6–8^ Several authors propose core needle biopsy as the sampling method of choice, as the complication rate has been shown to be 0–10%, compared with 16% for open biopsy.^9^ Open biopsy carries increased risks of wound breakdown, haematoma and infection, as well as increasing the risk of tumour seeding along biopsy tracts that are not removed during surgical resection.^9^ To minimize the risks of biopsy tract seeding, biopsy approach should coincide with the planned surgical incision site such that the tract is easily removed during any future surgical intervention.^10^ Occasionally, it can be difficult for the radiologist to obtain an appropriate tumour sample for diagnosis when limiting the biopsy approach to the surgical incision plane.^10^ At our institution, such difficult cases are always discussed with the multidisciplinary team.

Despite the first reported case of musculoskeletal tumour biopsy tract seeding in 1993, exact rates of occurrence are not yet clear.^7^ Recently, UyBico et al^11^ reviewed 363 cases of percutaneous musculoskeletal tumour biopsies and found no instances of needle tract seeding, suggesting that concern for iatrogenic seeding of musculoskeletal tumours may be more widespread than the evidence base to support it.

In view of the findings of our case and that of other cases of musculoskeletal tumour tract seeding, it may be appropriate that short-term post-biopsy imaging be performed to assess for evidence of biopsy tract seeding prior to definitive therapy, in instances where anatomic compartment breach may have occurred, whether histology yields a malignant or benign proliferative process such as chondroid tumour.^6–8^

## Learning points

1. Distinguishing periosteal chondroma from chondrosarcoma is challenging, as there is significant overlap of symptoms, imaging and histological features.
2. Lesion size is a good differentiator between periosteal chondroma and periosteal chondrosarcoma: lesions less than 3 cm in size are likely to be benign, whereas lesions greater than 7 cm are likely to be malignant.
3. Both benign and malignant cartilaginous lesions can breach extra-anatomical compartments following iatrogenic trauma, including biopsy, the approach of which should be confirmed with the treating tumour service.
4. Follow-up imaging pafter biopsy of cartilaginous lesions may be appropriate to assess for evidence of seeding in instances where anatomic compartment breach may have occurred, whether or not histology demonstrated a benign or malignant proliferative process.

## Ethics approval

Our institute’s Quality Assurance sub-committee of the Human Research Ethics department has reviewed this case and is agreeable to its publication.
